# Pilot Study on Visual Function and Fundus Autofluorescence Assessment in Diabetic Patients

**DOI:** 10.1155/2016/1287847

**Published:** 2016-02-10

**Authors:** Ana M. Calvo-Maroto, José J. Esteve-Taboada, Rafael J. Pérez-Cambrodí, David Madrid-Costa, Alejandro Cerviño

**Affiliations:** ^1^Optometry Research Group, Department of Optics & Optometry & Vision Sciences, University of Valencia, Burjassot, 46100 Valencia, Spain; ^2^Department of Ophthalmology, Oftalmar, Medimar International Hospital, 03016 Alicante, Spain

## Abstract

*Purpose*. Evaluate optimized fundus autofluorescence (FAF) imaging in early stages of diabetic retinopathy (DR) and relate findings with conventional colour fundus imaging and visual function in diabetic patients and control subjects.* Materials and Methods*. FAF and colour images were obtained using the CR-2 Plus digital nonmydriatic retinal camera in seven diabetic patients and thirteen control subjects. Visual-Functioning Questionnaire-25 (VFQ-25) and Diabetes Self-Management Questionnaire (DSMQ) were used to assess the quality of life and diabetes self-care. Contrast sensitivity function (CSF) was evaluated with the Vistech 6500 chart.* Results*. FAF and optimized-FAF imaging showed more retinal alterations related to DR than colour imaging. In diabetic patients, compatible signs with microaneurysms, capillary dilations, and haemorrhages were less numerous in colour imaging than optimized-FAF and FAF imaging in areas analysed. Control subjects at risk of developing DM showed more retinal pigment epithelium defects than those without risk in all retinal areas. Significant differences were not found in VFQ-25 and CSF between diabetic patients and control subjects.* Conclusions*. FAF and optimized-FAF imaging showed significant alterations related to DR not observed in colour imaging. FAF and optimized-FAF images could be a useful complementary tool for detecting early alterations associated with the development and progression of DR.

## 1. Introduction

Diabetic retinopathy (DR) is a common complication of diabetes mellitus caused by long-term damage to retinal microvasculature that implies visual impairment [1–3]. A combination of 35 studies determined that the overall prevalence of any DR was 34.6% among diabetic patients [4]. The diabetes-induced mechanism that contributes to development of DR remains understanding [1, 2].

Retinal pigment epithelium (RPE) and choroid are essential layers to maintain the normal metabolism and function of the retina. Any alteration of these layer functions implies a degeneration of photoreceptors, visual impairment, and even blindness [5].

The main substrate for lipofuscin formation in the RPE is the undegradable end products that results from the phagocytosis of photoreceptor outer segment located in RPE [6, 7]. In DR, lipofuscin contains numerous molecules mainly composed of peroxidation products from lipids and protein [8] and it could be used as an indicator of oxidative damage on the retina [9]. Thus, lipofuscin accumulation reflects the metabolic damage in RPE caused by disease [10].

The fundus autofluorescence (FAF) imaging is a noninvasive method that represents the distribution of lipofuscin in the RPE layer in vivo. This technique is based on the retinal capacity for light emission of a specific wavelength from natural fluorophores, mainly lipofuscin, in the RPE. This process occurs when these molecules are excited by suitable wavelength of light. The intensity of FAF depends on amount and distribution of lipofuscin [11, 12].

Some studies have shown specific FAF patterns for numerous ocular diseases such as diabetic macular oedema (DME) [6, 9, 13], cystoid macular oedema (CME) [14, 15], and age-related macular degeneration (AMD) [16, 17]. The results of these studies demonstrated that FAF could be helpful for reflecting the extent of retinal damage and monitoring the progression of disease [6, 7, 12, 16].

The present pilot study evaluates FAF in diabetic patients and control subjects using the CR-2 Plus AF nonmydriatic retinal camera (Canon Inc., Tokyo) with the aim of identifying the utility of optimized-FAF imaging in early detection of DR. Retinal alterations were also related with visual function, quality of life, and diabetes self-care management.

## 2. Material and Methods

### 2.1. Subjects

We studied 7 right eyes from 7 subjects with noninsulin dependent DM (4 men and 3 women) and 13 right eyes from 13 control subjects (9 men and 4 women). All patients provided informed written consent in accordance with the World Medical Association's Declaration of Helsinki before the procedures took place.

Inclusion criteria were clear ocular media allowing recording high quality colour and FAF imaging. Exclusion criteria for both diabetic patients and normal subjects were previous retinal photocoagulation and any retinal alteration affecting macula, such as AMD.

All diabetic patients were diagnosed with DM by a general practitioner from the National Health System.

The quality of life of diabetic and control groups was assessment by National Eye Institute Visual-Functioning Questionnaire-25 (VFQ-25) [18]. The reliability and validity of this questionnaire have been confirmed on patients with DR [19]. Diabetes Self-Management Questionnaire (DSMQ) [20] was administered in diabetic patients and a custom-made questionnaire to identify people at increased risk for undiagnosed diabetes [21].

Ocular examination included measurement of best-corrected visual acuity by Snellen chart, autorefractometer, slit-lamp biomicroscopy, contrast sensitivity function (CSF), colour fundus, and FAF imaging. Visual acuity (VA) was converted to the logarithm of the minimum of angle resolution [log⁡MAR] scale. Colour fundus and FAF imaging were recorded after fasting blood glucose levels measurement.

Fasting blood glucose levels were measured from 8.00 a.m. to 10.00 a.m. Glycosylated hemoglobin (HbA1c) levels were provided by diabetic patients.

### 2.2. Questionnaires

VFQ-25 consists of 25 vision-targeted questions divided into 11 vision-related subscales: General Vision rating (1), difficulty with near vision activities (3), difficulty with distance vision activities (3), limitations in social functioning due to vision (2), mental health symptoms due to vision (4), role difficulties due to vision (2), dependency on others due to vision (3), driving difficulties (3), limitations with peripheral vision (1), colour vision (1), and ocular pain (2). An additional single-item question is related to general health rating [18]. The scale of this questionnaire can vary between 0 (worst possible score) and 100 (best possible score).

DSMQ is a questionnaire designed to assess the self-care behavior related to the glycemic control. This questionnaire consists of 16 items divided into four subscales, Glucose Management, Dietary Control, Physical Activity, and Health-Care Use [20]. The scale transformed can vary between 0 and 10.

The new questionnaire developed to identify people at increased risk for undiagnosed diabetes is divided into three age ranges (20–44, 45–64, and 65 or plus years of age). The questions are related to body mass index and style of life (sedentary) for ranges of 20–44 and 45–64 years of age. For range of 65 or plus years of age, questions are related to body mass index (BMI) and familiar antecedents (father, mother, or brothers) and a question was about their baby weight in delivery in women [21].

### 2.3. Study Procedures

#### 2.3.1. CSF Measurements

CSF measurements were carried out with the Vistech 6500 chart (Vistech Consultants Inc.) which is a panel with 5 rows of 9 printed circular patches. The rows increase in spatial frequency from top to bottom of the chart, and on each row the contrast decreases from left to right. There are 5 spatial frequencies across the rows (1.5, 3, 6, 12, and 18 cycles per degree, resp.). The contrast decreases in increments of 0.12 log unit. The direction of the last patch correctly identified by the patient was recorded for each frequency. The patient was at 3 meters of chart and test was performed in a monocular way. The measurements were carried in photopic conditions (85 cd/m^2^).

#### 2.3.2. Colour Fundus and Fundus Autofluorescence Imaging: Evaluation and Image Acquisition

Colour and FAF images were recorded with CR-2 Plus digital nonmydriatic retinal camera (Canon, Tokyo). Colour images were obtained and evaluated for potential changes in the optic nerve, blood vessels, and macula. Following colour images, FAF images were taken using FAF mode (530–580 nm exciter filter and 640 nm barrier filter) in a dark room in order to prevent pupillary constriction. A single image was recorded per each eye.

The FAF images were saved as TIFF images and analysed by MATLAB 2013a software (MathWorks Inc., Natick, MA). All images were normalized and homogenized for a better visualization of retinal alterations and were called optimized-FAF images. Three images were obtained per eye, colour, FAF, and optimized-FAF images. All images were divided into four spatial quadrants that correspond to anatomical retinal quadrants: (1) upper temporal area, (2) lower temporal area, (3) upper nasal area, and (4) lower nasal area. The optic nerve was considered the center of the image (Figure 3), as it is commonly used for diagnostic staging of diabetic retinopathy in clinical practice [22].

These images were observed to find ophthalmic compatible signs with diabetic retinopathy such as microaneurysms, capillary dilation, hard exudates, and haemorrhages in diabetic patients.

In control subjects the observation was focused on retinal epithelial defects and capillary dilation findings.

### 2.4. Statistical Analyses

In this pilot study only right eye was considered for statistical analysis. Normality of data distribution for the different groups was determined using the Kolmogorov-Smirnov test. When parametric analysis was possible, the independent Student *t*-test was used for comparisons between diabetic patients and control subjects. When parametric analysis was not possible, the Mann-Whitney *U* test was used to assess the significance of such difference. Differences in ophthalmic signs of different areas between three images were assessed by Kruskal-Wallis test. To determine which of pairwise comparisons were responsible for the overall difference between groups, separate Mann-Whitney *U* tests were performed on each pairwise (3 tests), alpha used 0.05/3 = 0.0167.

Correlation coefficients (Pearson or Spearman) were used to assess the correlation between variables. We considered values of *p* < 0.05 to be statistically significant. All statistical analysis was performed using SPSS software (version 19, SPSS Inc.).

## 3. Results

The mean age of diabetic patients was 54.857 ± 10.254 years (range 45–71 years) and that of control subjects was 40.769 ± 11.763 years (range 25–68 years) (*p* = 0.014). Characteristics of diabetic and control groups are summarized in Table 1.

In the diabetic group, four patients were treated for high cholesterol values and two patients for high blood pressure values and two patients had problems with their thyroid gland. Within control group, one patient was being treated for high blood pressure and three showed cholesterol values higher than 200 mg/dL. The diabetic group did not show any complication.

The VFQ-25 questionnaire revealed that diabetic patients and control subjects presented similar visual function scores since differences were not statistically significant in each subscale (Table 1). In “General Health” subscale, control subjects showed higher scores (69.231 ± 18.125) than diabetic patients (64.286 ± 19.670) (*p* = 0.438). However, “General Vision” subscale and Visual Specific subscales (“Social Functioning,” “Mental Health,” “Role Difficulties,” and “Dependency”) of diabetic subjects were higher compared to control subjects (*p* > 0.05). Also “Sum Scale” was slightly higher in diabetic patients (92.26 ± 4.139) than control subjects (87.618 ± 13.352) but not statistically different (*p* = 0.817) (Table 1).

According to scoring guide of DSMQ where a cut-off score ≤ 6.0 in total score shows a suboptimal self-care, all diabetic patients had adequate self-care with a “Total Score” mean of 7.093 ± 0.853. “Glucose Management” subscale showed the highest score (8.885 ± 1.574) and “Physical Activity” showed the lowest score (4.600 ± 2.436) (Table 2).

The questionnaire used in control subjects to assess the risk in undiagnosed people to have diabetes showed that 53% of control subjects had an increased risk of suffering diabetes. Age, fasting glucose levels, and VA differences between control subjects with and without risk to develop DM were not statistically different.

**Figure 1:**
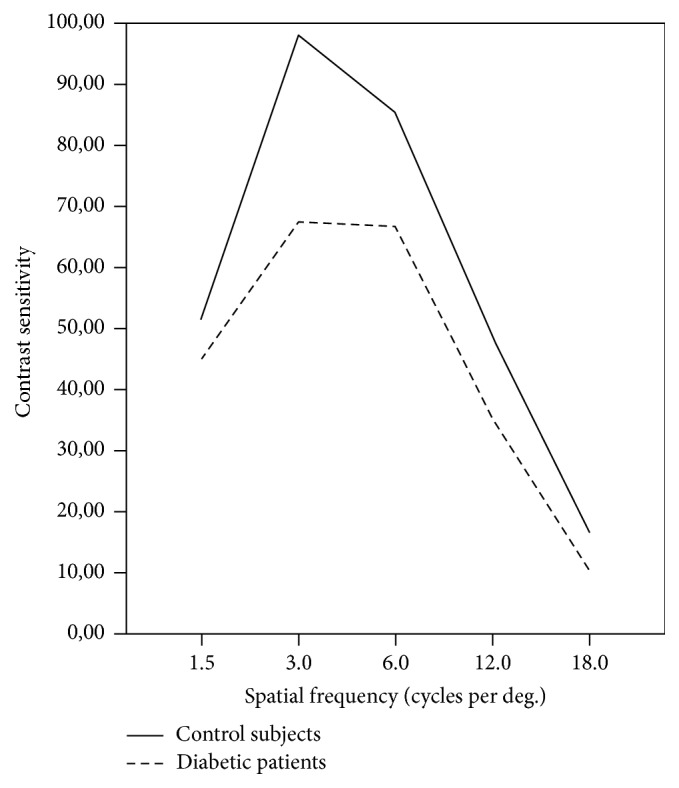
Contrast sensitivity function between diabetic patients (solid line) and control subjects (dashed/dotted line) (*p* > 0.05).

**Figure 2:**
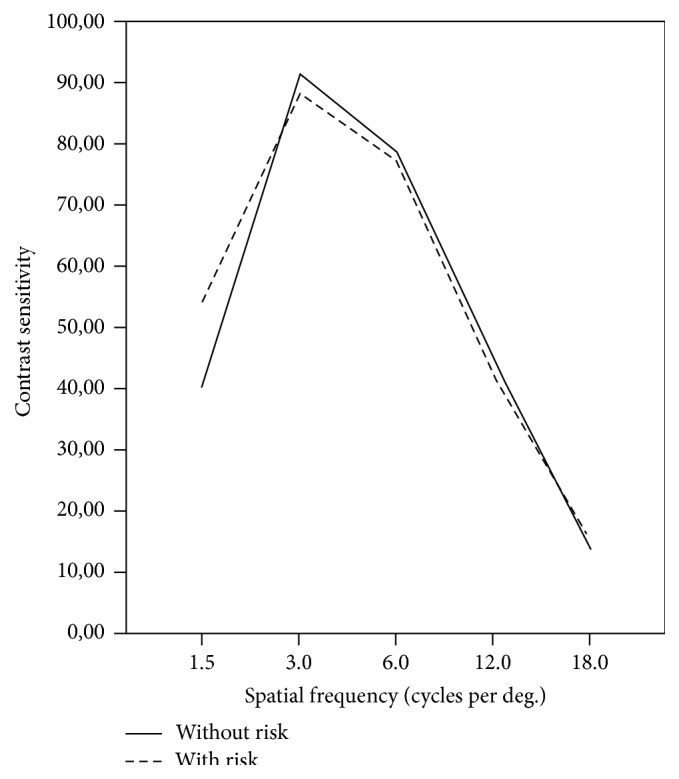
Contrast sensitivity function between control subjects with (dashed/dotted line) and without (solid line) risk of undergoing diabetes mellitus.

**Figure 3:**
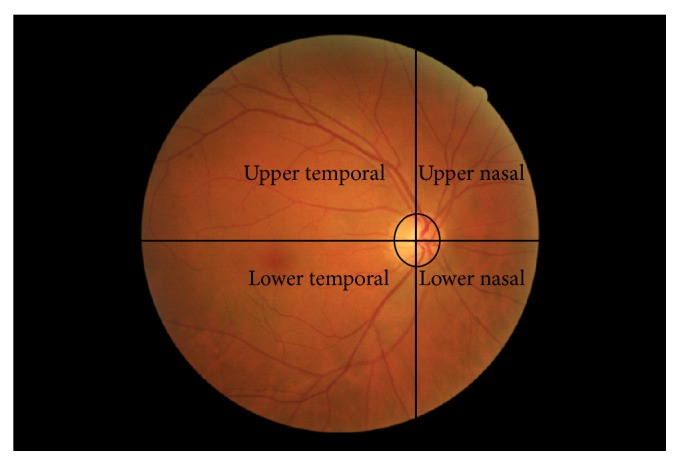
Division of fundus images into four quadrants centered at the optic nerve.

The CSF measurements revealed that differences at 1.5, 3, 6, 12, and 18 cycles per degree (cpd) were not statistically different between diabetic patients (45.0 ± 17.078, 67.429 ± 21.195, 66.714 ± 45.670, 35.286 ± 29.410, and 10.429 ± 9.253, resp.) and control subjects (51.154 ± 18.161, 98.077 ± 31.920, 85.385 ± 34.062, 48.846 ± 26.925, and 16.385 ± 8.560, resp.) (*p* > 0.05) (Figure 1). There were not statistically differences in CSF measures between control subjects with risk of developing DM compared to those without risk (*p* > 0.05) (Figure 2).

In VFQ-25 questionnaire, “General Health,” “General Vision,” and “Role Difficulties” subscales were inversely correlated with fasting glucose levels (*r* = −0.763, *p* = 0.002; *r* = −0.640, *p* = 0.018; *r* = −0.709, *p* = 0.007, resp.) in control subjects. In diabetic patients, “General Health” was inversely correlated with HbA1c levels (*r* = −0.784, *p* = 0.037).

No significant correlations were found between contrast sensitivity scores and age, fasting glucose level, and LogMAR VA in control and diabetic subjects. Diabetes duration and HbA1c levels were not significant correlated with contrast sensitivity scores in diabetic patients.

In diabetic patients, statistically significant differences of compatible signs with microaneurysms and capillary dilation between colour, FAF, and optimized-FAF images were found (Table 3). In all retinal areas, compatible signs with microaneurysms were less numerous in colour images compared with optimized-FAF and FAF images (Table 3). In colour versus FAF images, this decrease of ophthalmic signs observed was only statistically different for upper and lower temporal areas (*p* = 0.001 and *p* = 0.007, resp.). In colour versus optimized-FAF images, differences of compatible signs with microaneurysms were statistically different in all retinal areas (*p* < 0.0167). However, these ophthalmic signs were only statistically different at upper temporal area in FAF images compared to optimized-FAF images (*p* = 0.026) (Table 3).

Compatible signs with capillary dilation were more abundant in FAF and optimized-FAF images compared to colour images. However, differences were not statistically different between FAF and optimized-FAF images (*p* > 0.0167) (Table 3). Number of capillary dilations of FAF images was statistically higher at upper and lower temporal areas compared to colour images (*p* = 0.001). In colour versus FAF images, all retinal areas showed statistically significant differences in compatible signs with capillary dilations (*p* > 0.0167) except upper nasal area (*p* = 0.017). There were statistically significant differences in compatible signs with haemorrhages and hard exudates between colour, FAF, and optimized-FAF images (*p* > 0.05) (Table 3).

In control subjects, a number of epithelial defects and capillary dilations were statistically different between the three types of image (*p* < 0.001) (Table 4). Both retinal defects were statistically different between colour versus FAF images and colour versus optimized-FAF images (*p* < 0.0167) (Table 4). In FAF versus optimized-FAF images, epithelial defects were only statistically different at upper and lower temporal areas (*p* < 0.0167). However, statistically significant differences in capillary dilation number between FAF and optimized-FAF images were not found (*p* > 0.0167) (Table 4).

Based on DSMQ questionnaire, control subjects were divided into two groups, control subjects with risk to develop DM and those without risk. Although a number of ophthalmic epithelial signs observed in optimized-FAF images were higher than colour and FAF images, these signs were only statistically different at lower temporal area of colour images (*p* = 0.035). In FAF images, statistically significant differences between both groups were not found in any retinal area (*p* > 0.05). In optimized-FAF images, we only observed statistical differences at upper temporal area (*p* = 0.008). Related to capillary dilations, we only observed statistically significant difference at lower temporal area in colour images (*p* = 0.014). Statistical differences have not been found in FAF images and optimized-FAF images between both groups of control subjects (results not shown).

In diabetic patients, Spearman correlation revealed that compatible signs with microaneurysms observed at upper temporal and nasal areas in colour images were correlated with age (*r* = 0.845, *p* = 0.017; *r* = 0.905, *p* = 0.005, resp.) and glucose levels were correlated with lower temporal area (*r* = 0.919, *p* = 0.003). In FAF images, HbA1c levels were correlated with upper nasal area (*r* = 0.955, *p* = 0.001) and diabetes duration with lower nasal area (*r* = 0.827, *p* = 0.022). In optimized-FAF images, diabetes duration was correlated with upper and lower nasal areas (*r* = 0.757, *p* = 0.049; *r* = 0.811, *p* = 0.027).

Compatible signs with capillary dilation were only correlated with HbA1c levels in upper nasal area (*r* = 0.757, *p* = 0.049), and with glucose levels in lower nasal area (*r* = 0.757, *p* = 0.049), in optimized-FAF images. In control subjects, Pearson correlation showed that epithelial defects observed at upper nasal area were correlated with age (*r* = 0.613, *p* = 0.026) in FAF images. In optimized-FAF images, age was correlated with epithelial defects found at upper temporal area (*r* = 0.587, *p* = 0.035).

**Figure 4:**
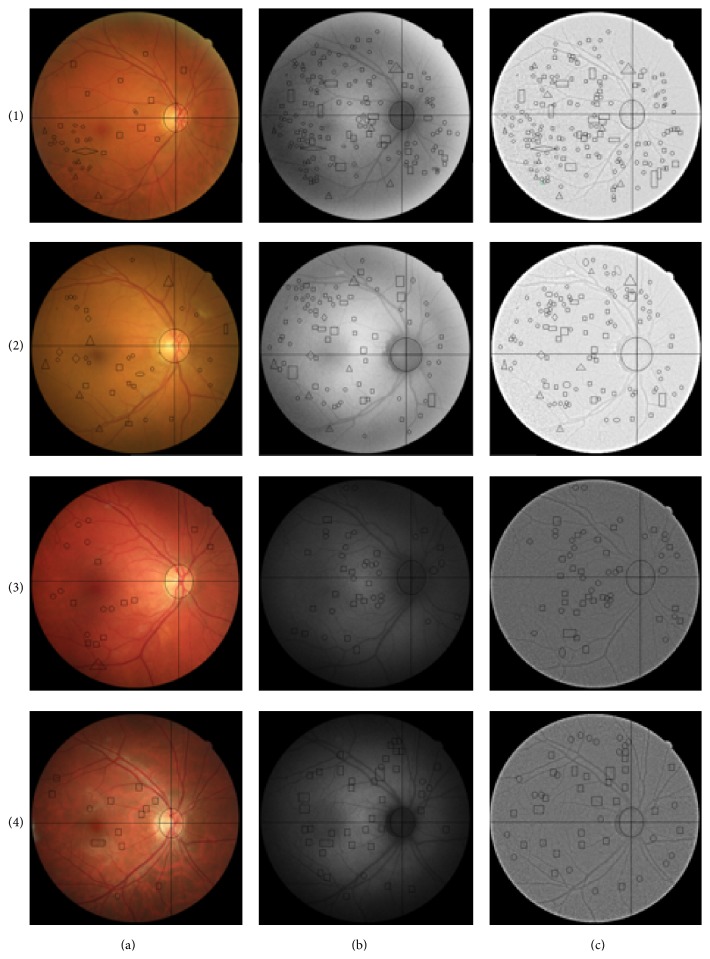
Colour fundus imaging (a), FAF imaging (b), and optimized-FAF (c) of (1) insulin dependent diabetes mellitus patient, diabetes duration 18 years; (2) noninsulin dependent diabetes mellitus patient, diabetes duration 30 years; (3) control subject with risk of developing diabetes mellitus; (4) control subject without risk of developing diabetes mellitus. Signs compatible with microaneurysms were represented by circle form, capillary dilation, haemorrhages, and hard exudates by square, triangle, and rhombus forms, respectively.

Figure 4 shows colour, FAF, and optimized-FAF images of diabetic patients and control subjects. FAF and optimized-FAF images of diabetic patients revealed retinal alterations while colour imaging did not present such alterations.

## 4. Discussion

The FAF is a noninvasive technique utilized mainly for detecting changes in metabolic activity at the RPE and it has been used in retinal diseases, such as AMD, DME, and other retinal diseases to develop typical imaging features and to determine the extent of retinal damage [6–17].

DR is a common microvasculature complication in diabetic patients characterized by the presence of microaneurysms at the earliest stage, known as mild nonproliferative diabetic retinopathy (NPDR) until the development of neovascularization and/or vitreous/preretinal haemorrhage at the proliferative diabetic retinopathy (PDR), the latest stage of this complication. Several pathways such as polyol accumulation, advanced glycation end products (AGEs), oxidative stress, and activation of protein kinase C (PKC) have been proposed as being responsible of microvasculature damage [2].

In this pilot study we assessed FAF images using CR-2 Plus digital nonmydriatic retinal camera (Canon, Tokyo) in diabetic patients and control subjects with the aim of detecting early retinal signs of DM before impacting at the visual quality. We also studied visual function of both groups, self-care of diabetes and risk of developing diabetes, by the use of questionnaires.

Diabetic patients showed significantly higher fasting glucose levels than control subjects (*p* < 0.001) (Table 1). Diabetic subjects presented stable blood glucose levels (<200 mg/dL) and HbA1c levels revealed a good glycaemic control (7.214%) (Table 1). Thus, according to DSMQ all diabetic patients had adequate self-care of DM (Table 2) (“Total Score” > 6) [20].

53% of control subjects had a risk to develop diabetes according to the new questionnaire developed to identify undiagnosed people of diabetes [21], due to lack of practical exercise and/or high values of BMI.

Relative to visual function, several studies have analysed VFQ-25 in diabetic patients [23, 24]. In these studies, determining that VFQ-25 is a better measure of visual function state in diabetic patients with DR than VA measure, due to degree of anxiety, fear, and mental anguish related to DR presence, was not assessed by VA measure. The progression of DR was related with a decrease of “Visual-Specific Mental Health” [23, 24], “Visual-Specific Role Difficulties,” “Visual-Specific Dependency,” and “driving” subscales [23]. However, in this study there are not differences in LogMAR VA, subscales scores, and “Sum Scale” of VFQ-25 between diabetic patients and control subjects (Table 1). This lack of significant differences between both of groups could be because our patients had been well controlled and these patients did not show retinal alterations related to advanced stage of DR.

In this study, statistical correlations between LogMAR VA and VFQ-25 subscales were not found in diabetic patients nor control subjects. Although we found that fasting glucose levels were inversely correlated with “General Health” (*r* = −0.763,  *p* = 0.002), “General Vision” (*r* = −0.640, *p* = 0.018), and “Role Difficulties” (*r* = −0.709, *p* = 0.007) subscales in control subjects, in diabetic controls, HbA1c levels were inversely correlated with “General Health” subscale (*r* = −0.784, *p* = 0.037).

In previous studies, contrast sensitivity scores were reduced in diabetic patients without retinopathy and it has been determined that contrast sensitivity measure could represent early retinal dysfunctions in diabetic patients without signs of DR [25–28]. In this study, spatial frequency scores of diabetic patients were lower than control subjects, but not statistically different (*p* < 0.05) (Figure 1). In addition to diabetes-related changes, this decrease could be explained partly by ageing because of a statistical difference in age between diabetic patients and control subjects.

Misra et al. in their study found an inversed statistically significant correlation between contrast sensitivity and LogMAR VA in diabetic patients without signs of DR, and they also demonstrate that HbA1c levels have a significant effect association with contrast sensitivity [25]. However, we did not found statistical correlations between LogMAR AV, HbA1c levels, and contrast sensitivity frequencies in diabetic patients. These differences could be due to a difference in diabetes duration; although in study of Misra et al. this variable is unknown, it is possible that their patients had duration of diabetes higher than our patients.

In control subjects with and without risk of developing diabetes spatial frequencies scores were similar, so there were not statistical differences at spatial frequencies (Figure 2). This lack of statistic difference between control subjects could be due to absence of early ocular alterations related with diabetes.

In the FAF pattern of healthy eyes, the optic nerve head appears dark due to absence of lipofuscin, retinal vessels present a reduction of FAF signal due to absorption by blood and in the macular area, and FAF signal is also reduced especially around the fovea due to absorption of macular pigment, such as lutein, zeaxanthin, and other pigments [7]. In DR, the mechanism of accumulation of lipofuscin in the retina is different from the other retinal diseases such as AMD [7]. In DR, age-related accumulation of lipofuscin in RPE, due to phagocytosis of outer segment photoreceptor, is not important in this retinal disease [29]. But, lipofuscin contains a large number of different products of peroxidation of lipids and proteins. So lipofuscin is thought as an indicator of oxidative damage in the retina [9]. Xu et al. showed that accumulation of lipofuscin was greater in microglia than in the RPE in this retinal disease. The pathophysiologic process of DR activates microglia; this activation allows the oxidation of proteins and lipids and therefore the accumulation of lipofuscin granules in the microglia in the development of diabetes [30].

In this study FAF imaging was normalized and homogenized for a better visualization of retinal alterations and was compared to colour fundus images. We observed that all compatible signs with DR were statistically different between colour, FAF, and optimized-FAF images (Table 3) (Figure 4). In FAF and optimized-FAF images, we observed hypofluorescent granules that could be compatible signs with microaneurysms. Thus, we observed that these signs were statistically more numerous in optimized-FAF than colour images (*p* < 0.0167). Related to compatible signs with capillary dilation, both temporal zones (upper and lower areas) showed statistically significant differences between colour and optimized-FAF images (*p* < 0.0167). However, compatible signs with haemorrhages and hard exudates were not found statistically different between colour, FAF, and optimized-FAF images (*p* > 0.05), although a number of hard exudates were slightly higher in colour images compared with FAF and optimized-FAF images. Hard exudates were identified as hyperfluorescent granules in FAF and optimized-FAF images. So compatible signs with microaneurysms and capillary dilation were more evident in FAF and optimized-FAF images compared with colour images but hard exudates were more difficult in their identification in FAF and optimized-FAF images. Also optimized-FAF images showed compatible alterations with DR not visible in colour fundus, such as microaneurysms and capillary dilations. Xu et al. showed the pathophysiologic process of DR active microglia and therefore formation of lipofuscin granules [30]. So we hypothesized that these alterations observed in optimized-FAF images could show early oxidative damage before formation of DR signs.

Although control subjects with risk to develop diabetes mellitus showed a large amount of epithelial defects compared to those without risk, these alterations were statistically different at lower temporal area of colour images and at upper temporal area of optimized-FAF images. Capillary dilations numbers were only statistically different at upper temporal area in colour images (*p* = 0.008) (Table 4). In control subjects with risk of developing DM, these epithelial alterations were more predominant in temporal areas and could correspond to local defects of RPE.

This study has limited sample size as main limitation, but the other limitation was that questionnaires were not validated in Spanish language. Therefore, all conclusions must be interpreted with care, as they need further confirmation in larger sample studies. In this study, we have compared FAF in colour fundus imaging, although future comparative studies with other imaging models, such as optical coherence tomography (OCT), could also be significant for DR diagnosis.

In summary, this study shows visual function, contrast sensitivity, FAF, and colour imaging in diabetic patients and control subjects. Diabetic patients had a good glycaemic control; however, we found that FAF imaging revealed alterations not visible in colour imaging that could be a sign of diabetes progression. Visual function and contrast sensitivity score were not statistically different between diabetic patients and control subjects. Although there is difficulty with image interpretation due to absence of protocols and measure system, in this study we considered that FAF and optimized-FAF images could be a complementary tool for detecting early signs of DR and for monitoring DM. Further studies are still needed to confirm these results and the exact mechanism of FAF changes in DM.

## Figures and Tables

**Table 1:** Descriptive statistics and scoring Visual-Functioning Questionnaire-25 (VFQ-25) of the diabetic patients and control subjects.

	Diabetic patients (*n* = 7)		Control subjects (*n* = 13)		*p* value^§^
	Mean ± SD	Range	Mean ± SD	Range	
Sex	4 M, 3 F	—	9 M, 4 F	—	
Age (yr)	54.857 ± 10.254	45–71	40.769 ± 11.763	25–68	0.014
LogMAR AV	0.045 ± 0.086	0–0.222	0.007 ± 0.027	0–0.097	0.438
Fasting glucose levels (mg/dL)	147 ± 16.653	130–181	95.461 ± 8.402	84–112	<0.001
HbA1c levels (%)	7.214 ± 1.120	6.0–9.60	—	—	
Diabetes duration (months)	182.143 ± 140.741	3–360	—	—	
General Health	64.286 ± 19.670	50–100	69.231 ± 18.125	25–100	0.438
General Vision	74.286 ± 9.759	60–80	70.770 ± 15.525	40–80	0.817
Ocular pain	94.643 ± 9.835	66.67–100	81.731 ± 16.626	50–100	0.097
Distance vision activities	92.829 ± 8.908	75–100	89.098 ± 14.184	50–100	0.588
Near vision activities	83.096 ± 11.644	66.67–100	91.665 ± 16.316	41.67–100	0.311
Visual-Specific Social Functioning	98.214 ± 4.724	87.50–100	95.192 ± 9.599	75–100	0.699
Visual-Specific Mental Health	83.929 ± 9.449	68.75–93.75	81.731 ± 24.535	6.25–100	0.536
Visual-Specific Role Difficulties	92.857 ± 12.199	75–100	86.538 ± 20.704	37.50–100	0.588
Visual-Specific Dependency	100	—	92.948 ± 19.199	33.33–100	0.588
Driving	79.763 ± 36.279	0–100	73.071 ± 35.381	0–100	0.536
Colour vision	100	—	98.077 ± 6.934	75–100	0.817
Peripheral vision	96.429 ± 9.450	75–100	88.461 ± 19.406	50–100	0.536
Sum Scale	92.26 ± 4.139	86.44–96.54	87.618 ± 13.352	51.55–97.61	0.817

^§^Mann-Whitney *U* test.

LogMAR AV: Visual Acuity Logarithm of the Minimum of Angle Resolution; HbA1c: Glycated haemoglobin.

**Table 2:** Scoring of Diabetes Self-Management Questionnaire (DSMQ) in diabetic patients.

Patients	Glucose Management	Dietary Control	Physical Activity	Health-Care Use	Total score
1	6.66	7.5	8.88	5.55	7.083
2	10	6.66	3.33	7.77	7.5
3	6.66	6.66	2.22	6.66	5.625
4	10	9.16	2.22	10	8.125
5	10	7.5	4.44	8.88	7.916
6	8.88	7.5	4.44	8.88	6.739
7	10	0	6.67	2.22	6.667

Mean ± SD	8.886 ± 1.574	6.426 ± 2.953	4.6 ± 2.435	7.137 ± 2.633	7.093 ± 0.853

**Table 3:** Distribution of retinal alterations observed in colour, FAF, and optimized-FAF images in diabetic patients.

Compatible signs	Image type			*p* value^a^	*p* value post hoc test^b^		
	Colour	FAF	Optimized-FAF		Colour versus FAF	Colour versus optimized-FAF	FAF versus optimized-FAF
Microaneurysms							
Upper temporal area	4.43 ± 3.82	28.43 ± 6.29	42.57 ± 12.98	<0.001	0.001	0.001	0.026
Lower temporal area	7.28 ± 3.95	23.43 ± 8.54	35.71 ± 11.98	0.001	0.007	0.001	0.073
Upper nasal area	1.14 ± 1.46	4.14 ± 2.27	12.14 ± 9.12	0.005	0.017	0.004	0.097
Lower nasal area	1.86 ± 2.27	9.00 ± 5.60	13.71 ± 8.48	0.007	0.026	0.002	0.318
Capillary dilation							
Upper temporal area	3.43 ± 2.15	22.71 ± 5.74	19.86 ± 2.34	0.001	0.001	0.001	0.209
Lower temporal area	3.86 ± 1.86	19.86 ± 7.42	16.57 ± 3.55	0.001	0.001	0.001	0.383
Upper nasal area	0.43 ± 0.53	2.71 ± 1.70	5.00 ± 4.28	0.02	0.017	0.017	0.535
Lower nasal area	0.43 ± 0.53	4.71 ± 3.95	4.14 ± 4.06	0.01	0.004	0.017	0.805
Hemorrhages							
Upper temporal area	1.14 ± 1.46	2.14 ± 2.11	2.29 ± 2.50	0.516	—	—	—
Lower temporal area	3.00 ± 1.63	2.71 ± 1.60	2.86 ± 1.95	0.914	—	—	—
Upper nasal area	—	—	—	—	—	—	—
Lower nasal area	0.43 ± 0.79	0.43 ± 0.79	0.43 ± 0.79	1	—	—	—
Hard exudates							
Upper temporal area	1.43 ± 2.23	0.71 ± 1.50	1.14 ± 2.61	0.819	—	—	—
Lower temporal area	1.57 ± 2.93	1.28 ± 2.98	1.28 ± 2.98	0.823	—	—	—
Upper nasal area	—	—	—	—	—	—	—
Lower nasal area	—	—	—	—	—	—	—

^a^Kruskal-Wallis test; ^b^Mann-Whitney *U* test; FAF: fundus autofluorescence.

**Table 4:** Distribution of retinal alterations observed in colour, FAF, and optimized-FAF images in control subjects.

	Image type			*p* value	*p* value post hoc test		
	Colour	FAF	Optimized-FAF		Colour versus FAF	Colour versus optimized-FAF	FAF versus optimized-FAF
Epithelial defects							
Upper temporal area	1.92 ± 2.06	10.08 ± 4.60	19.61 ± 7.69	<0.001^a^	<0.001^c^	<0.001^c^	<0.001^c^
Lower temporal area	1.15 ± 1.28	9.61 ± 6.65	18.77 ± 9.04	<0.001^a^	<0.001^c^	<0.001^c^	0.008^c^
Upper nasal area	0.85 ± 1.40	3.08 ± 2.29	5.61 ± 3.69	<0.001^b^	0.007^d^	<0.001^d^	0.048^c^
Lower nasal area	0.92 ± 1.60	3.08 ± 2.06	5.61 ± 3.20	<0.001^b^	0.01^d^	<0.001^d^	0.026^c^
Capillary dilation							
Upper temporal area	3.85 ± 2.91	13.77 ± 4.21	12.23 ± 3.63	<0.001^a^	<0.001^c^	<0.001^c^	0.328^c^
Lower temporal area	4.15 ± 2.73	13.15 ± 7.20	13.00 ± 4.08	<0.001^a^	<0.001^c^	<0.001^c^	0.947^c^
Upper nasal area	0.69 ± 1.18	3.08 ± 1.98	3.15 ± 1.62	<0.001^a^	0.001^c^	<0.001^c^	0.915^c^
Lower nasal area	0.54 ± 0.97	2.69 ± 2.21	3.31 ± 1.97	<0.001^b^	0.001^d^	<0.001^d^	0.462^c^

^a^One-way ANOVA test; ^b^Kruskal-Wallis test; ^c^unpaired *t* test; ^d^Mann-Whitney *U* test; FAF: fundus autofluorescence.
